# Ketamine Alleviates Postoperative Depression-Like Symptoms in Susceptible Mice: The Role of BDNF-TrkB Signaling

**DOI:** 10.3389/fphar.2019.01702

**Published:** 2020-02-06

**Authors:** Shan Li, Xiaoxiao Luo, Dongyu Hua, Yue Wang, Gaofeng Zhan, Niannian Huang, Riyue Jiang, Ling Yang, Bin Zhu, Xianglin Yuan, Ailin Luo, Chun Yang

**Affiliations:** ^1^ Department of Anesthesiology, Tongji Hospital, Tongji Medical College, Huazhong University of Science and Technology, Wuhan, China; ^2^ Department of Internal Medicine, The Third Affiliated Hospital of Soochow University, Changzhou, China; ^3^ Department of Oncology, Tongji Hospital, Tongji Medical College, Huazhong University of Science and Technology, Wuhan, China; ^4^ Department of Anesthesiology and Perioperative Medicine, The First Affiliated Hospital of Nanjing Medical University, Nanjing, China

**Keywords:** postoperative depression, ketamine, BDNF-TrkB signaling, elderly, sex difference

## Abstract

Patients are more likely to suffer from central nervous system (CNS) complications after anesthesia and surgery. However, postoperative depression (POD) has not yet received sufficient attentions, and its pathogenesis and therapeutic strategies remain poorly understood. We here aimed to investigate whether brain derived neurotrophic factor (BDNF)-tropomyosin-related kinase B (TrkB) signaling plays an important role in POD. BDNF-TrkB signaling was altered in brain and peripheral tissues, including medial prefrontal cortex (mPFC), hippocampus, liver, and muscle, among control, POD susceptible, and resilient groups. Additionally, we demonstrated that 7,8-dihydroxyflavone (7,8-DHF), a TrkB agonist, could exert its pharmacologic property to alleviate POD-like symptoms. More importantly, ketamine, a non-competitive N-methyl-D-aspartic acid (NMDA) receptor antagonist, also has significant antidepressant effects in POD model, associating with the improving effects on levels of BDNF-TrkB signaling in brain and peripheral tissues. Interestingly, the beneficial effects of ketamine on POD-like symptoms are fully attenuated by a TrkB antagonist. These findings suggest that abnormal expressions of BDNF-TrkB signaling in brain and peripheral tissues are implicated in the pathogenesis of POD, and that therapeutic agents targeting BDNF-TrkB, particularly ketamine, could favor the beneficial effects for POD.

## Introduction

Inevitably everyone will encounter a condition which needs effective treatment involving in surgery and anesthesia. In addition, approximately 15 % of patients undergoing various surgeries are at higher risk of central nervous system (CNS) complications, leading to 80 % of all deaths during perioperative period (Ghoneim and O'Hara, 2016). Various stressors during the surgery, such as anesthetic agents, anxiety, pain, and surgical stress, might greatly contribute to abnormal behaviors postoperatively, including anxiety, depression, chronic pain, and cognitive dysfunction (Samuelsson et al., 2007; Costigan et al., 2009; Patel and Sun, 2009). In a recent study, nearly half of orthopedic patients were reported to be depressed postoperatively (Nickinson et al., 2009). And also, meta-analysis of randomized controlled trials provide a strong evidence that perioperative depression was significantly correlated with the early and later morality in patients undergoing cardiac surgery (Jiang et al., 2016). It has been demonstrated that patients with postoperative depression (POD) are prone to suffer from increased incidences of infections, cognitive dysfunction, and mortality from cancer postoperatively (Ghoneim and O'Hara, 2016). However, little academic attentions have been focused on POD, and more importantly, its pathogenesis and therapeutic mechanisms remain unknown.

Ketamine, a commonly-used anesthetic agent, shows rapid-acting and sustained antidepressant effect in the treatment-resistant patients with major depressive disorder (MDD) and bipolar disorder (BD) (Machado-Vieira et al., 2009; McGirr et al., 2015; Machado-Vieira et al., 2017). Interestingly, ketamine also exhibits rapid and sustained anti-suicidal ideation in treatment-resistant patients with MDD or BD (Zarate et al., 2006; DiazGranados et al., 2010; Zarate et al., 2012). Ketamine's anti-depressant effects were reported to be involved in the increased level of brain derived neurotrophic factor (BDNF), which is closely associated with the pathogenesis of POD (Jiang et al., 2016). This suggests that ketamine might reduce the incidence of POD and enhance resilience to the stress caused by surgery and anesthesia. In recent studies, the effects of ketamine on the prevention and treatment of POD in clinical researches were controversial. It was reported that intraoperative administration of low dose ketamine could improve depressive mood in patients undergoing orthopedic surgery on the postoperative day 1 and 5 (Kudoh et al., 2002; Jiang et al., 2016). However, intraoperative application of ketamine in parturients in the caesarean section and older adults in major surgery didn't have a preventive effect on postpartum depression (Xu et al., 2017; Mashour et al., 2018). We can speculate that the benefits of ketamine on POD lie on a wide range of factors, including age, sex, surgery type, anesthesia protocol, antidepressants use, and depression measurement method etc. Thus a standard and more generalized preclinical research is required to elucidate the potential etiology of POD and explore the effect of ketamine on POD.

BDNF, a protein mainly synthesized in the neurons, is widely distributed in the CNS. It plays a pivotal role in the neuronal survival, differentiation, growth, and development. Increasing evidence shows that BDNF exerts its physiologically beneficial effects mainly via binding to its specific receptor tropomyosin related kinase B (TrkB) (Harward et al., 2016; Song et al., 2017). Decreased levels of BDNF-TrkB signaling pathway were often reported in depressed subjects, and up-regulation of BDNF-TrkB via pharmacological agents in the prefrontal cortex (PFC) and hippocampus can confer resilience to inescapable stress and alleviate depression symptom (Ren et al., 2016; Kato et al., 2018). More importantly, TrkB-dependent hippocampal neurogenesis and differentiation was closely involved in the ketamine's rapid and sustained antidepressant effects (Ma et al., 2017). Based on these, we tend to explore the possibility whether ketamine has facilitating effects on POD and whether BDNF-TrkB signaling is involved in the pathogenesis and therapeutic mechanism of POD.

We here classified the mice underwent anesthesia and surgery into POD susceptible, resilient, and undetermined phenotypes, and attempted to determine the role of BDNF-TrkB signaling in POD. Furthermore, we designed to observe the effects of ketamine on POD-like symptoms and to elucidate whether BDNF-TrkB signaling is involved in ketamine exerting pharmacologically improving effects on POD-like symptoms.

## Materials and Methods

### Animals

Two month-old male/female C57BL/6 mice (body weight 20–25 g) and 16-month-old male C57BL/6 mice (body weight 28–31 g) in the experiment were purchased from the Animal Center of Tongji Hospital. A total of 251 mice were enrolled and were randomly divided into groups. Animals were acclimated to the environmental conditions for 7 days before the experiment. Animals were housed in controlled temperature (22 ± 2°C) and 12 h light/dark cycles (lights on 8:00) with food and water ad libitum. All experimental protocols and animal handing procedures were carried out in strict accordance with the recommendations in the Guide for the Care and Use of Laboratory Animals. The experimental protocol was approved by the Experimental Animal Committee of Tongji Hospital, Tongji Medical College, Huazhong University of Science and Technology (Wuhan, China).

### Experiment Design

**Figure 1:**
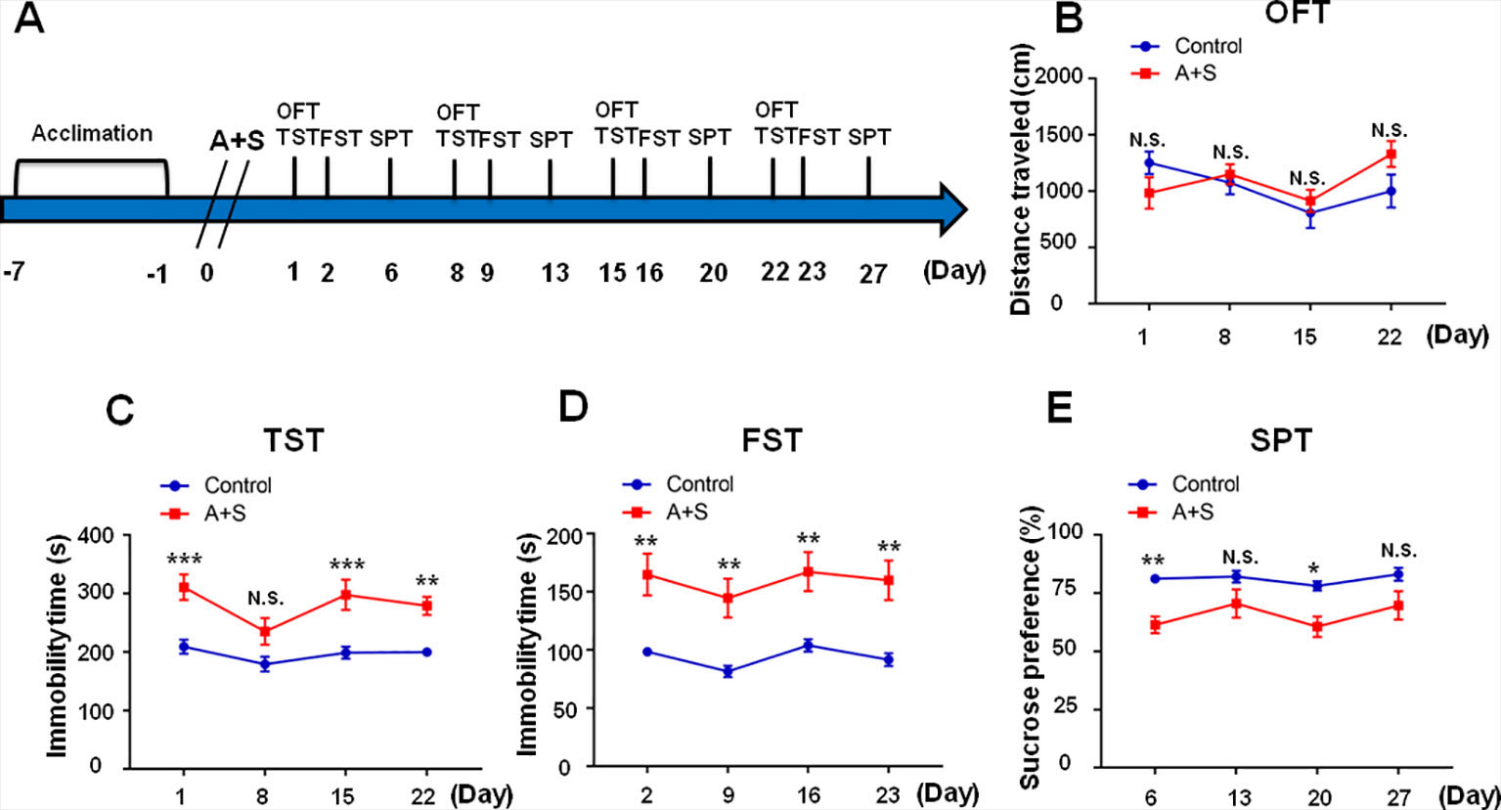
Effects of anesthesia and surgery on depression-like symptoms in OFT, TST, FST, and SPT. The results of OFT, TST, FST, and SPT in control and A+S mice. **(A)** The schedule of the experiment. A+S was performed on day 0 after acclimation. OFT and TST were measured on day 1, 8, 15, and 22 after A+S, respectively. FST was performed on day 2, 9, 16, and 23 after A+S, respectively. SPT was performed on day 6, 13, 20, and 27 after A+S, respectively. **(B)** OFT [time: *F*_(3,__54)_ = 2.679, *P* > 0.05; group: *F*_(1,__18)_ = 0.3479, *P =* 0.5627; interaction: *F*_(3,__54)_ = 1.887, *P* = 0.1427] in control and A+S groups. **(C)** TST [time: *F*_(3,__54)_ = 3.151, *P* = 0.0322; group: *F*_(1,__18)_ = 65.29, *P* < 0.001; interaction: *F*_(3,__54)_ = 0.6903, *P* = 0.5619] in control and A+S groups. **(D)** FST [time: *F*_(3,__54)_ = 1.343, *P* = 0.27; group: *F*_(1,__18)_ = 41.97, *P* < 0.001; interaction: *F*_(3,__54)_ = 0.02221, *P* = 0.9955] was measured in control and A+S groups. **(E)** SPT [time: *F*_(3,__54)_ = 1.76, *P* = 0.1659; group: *F*_(1,18)_ = 23.76, *P* < 0.001; interaction: *F*_(3,__54)_ = 0.4846, *P* = 0.6944] was measured in control and A+S groups. Data are shown as mean ± SEM (n = 10). *P < 0.05, **P < 0.01, ***P < 0.001. A+S, anesthesia and surgery; FST, forced swimming test; N.S., not significant; OFT, open field test, SPT, sucrose preference test; TST, tail suspension test.

**Figure 2:**
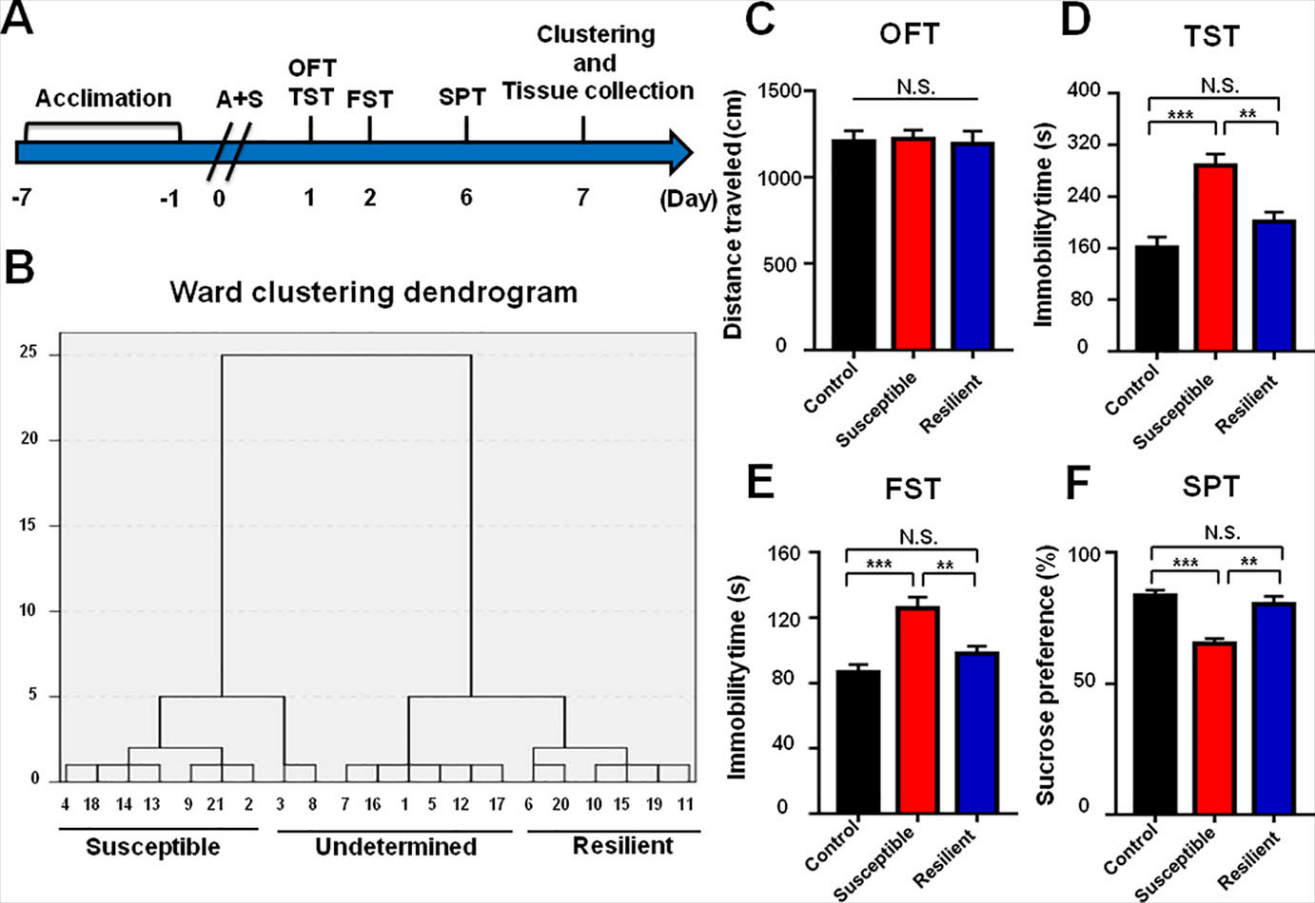
Comparisons of behavioral results in control, POD susceptible, and resilient groups within 1 week postoperatively. **(A)** The schedule of A+S and behavioral tests. Behavioral tests, including OFT, TST, FST, and SPT were performed on day 1, 2, and 5 after A+S, respectively. Tissue samples were collected on day 7. **(B)** Dendrogram of hierarchical clustering analysis. A total of 21 mice after A+S were divided into depression susceptible, resilient, and undetermined groups by TST, FST, and SPT results of hierarchical clustering analysis. **(C)** OFT [*F*_(2,__17)_ = 05446, *P* = 0.9472]. **(D)** TST [*F*_(2,__17)_ = 15.66, *P* < 0.001]. **(E)** FST [*F*_(2,__17)_ = 12.94, *P* < 0.001]. **(F)** SPT [*F*_(2,__17)_ = 17.08, *P* < 0.001]. Data are shown as mean ± S.E.M. (n = 6~7). **P < 0.01 or ***P < 0.001. A+S, anesthesia and surgery; FST, forced swimming test; N.S., not significant; OFT, open field test; POD, postoperative depression; SPT, sucrose preference test; TST, tail suspension test.

As shown in the **Figure 1**, mice were acclimated to the environment for 7 days, and then abdominal surgery was performed on day 0. Open field test (OFT) and tail suspension test (TST) were measured on day 1, 8, 15, and 22 after anesthesia and surgery (A+S), respectively. TST was performed 30 min after OFT. Forced swimming test (FST) was performed on day 2, 9, 16, and 23 after A+S, respectively. Sucrose preference test was performed on day 6, 13, 20, and 27 after A+S, respectively. A single dose of ketamine (10 mg/kg) or 7,8-DHF(10 mg/kg) was intraperitoneally injected 30 min before OFT to investigate its effects on depression-like behaviors (**Figures 4A** and **6A**). TST, FST, and SPT were used to perform cluster analysis on day 7 to divide mice in A+S group to depression susceptible, resilient, and undetermined clusters (**Figures 2A**, **7A**, **8A**, **9A**). According to a previous study, depression susceptible mice were treated with a single dose of ANA-12 (0.5 mg/kg) and ketamine (10 mg/kg) simultaneously after clustering (Sun et al., 2016) (**Figure 7A**). Mice were anesthetized in a cylinder which was full with isoflurane, and then medial prefrontal cortex (mPFC), hippocampus of brain, and liver, skeletal muscle were collected (**Figures 2A**, **4A**). Tissue samples were stored in −80°C before Western Blot analysis.

### Drug Administration

7,8-Dihydroxyflavone (7,8-DHF, ab120996, Abcam, UK) and N2-(2-[(2-oxoazepan-3-yl)amino]carbonylphenyl)benzo[b]thiophene-2-carboxamide (ANA-12, HY12497, Medchem Express, USA) were prepared in 17% dimethyl sulfoxide (DMSO) in phosphate-buffered saline. Ketamine hydrochloride (Fujian Gutian Pharmaceutical Co., Ltd. Fujian, China) was diluted by saline. Vehicle was referred to 17% DMSO in phosphate-buffered saline. The doses of ketamine (10 mg/kg), 7,8-DHF (10 mg/kg), ANA-12 (0.5 mg/kg), and vehicle or saline (10 ml/kg) were selected as reported previously (Yang et al., 2013; Zhang et al., 2014; Fang et al., 2018). All the compounds were intraperitoneally administered.

### Anesthesia and Surgery

Abdominal surgery was performed as our previous study (Zhang et al., 2018). Briefly, all mice were randomly assigned into A+S group or control group. Mice in A+S group were placed in a transparent acrylic chamber which was full with 1.4% isoflurane and 100% oxygen for 30 min, and then the heads of mice were put into a mask to maintain 1.4% isoflurane and 100% oxygen, monitoring the concentration of isoflurane with an infrared probe (OhmedaS/5 Compact; Datex‐Ohmeda, Louisville, KY) during the surgery period. After shaving and sterilizing the surgical site, a 0.5 cm incision was made through the skin and muscle wall of the ventral midline. A sterile probe was then inserted into the body cavity to gently manipulate the internal organs for 5 min, after that small intestine exteriorization for 10 min. Finally, abdominal skin and peritoneum were sutured using 5-0 Vicryl thread. The mice were then put back to the chamber for up to 2 h anesthesia. Compound lidocaine cream was given to the incision for analgesia three times daily for 3 consecutive days postoperatively. Mice in control group were placed in a similar transparent acrylic chamber with a mix of 70%O_2_/30%N_2_ for 2 h. Heating blanket was used to keep the rectal temperature of all mice at 37 ± 0.5°C during the surgery and postoperative recovery period.

### Behavioral Tests

#### OFT

After being applied with the compound lidocaine cream, mice were placed in the center of the Plexiglas chamber (40 × 40 × 40 cm) alone for 5 min, and the total distance traveled (meters) was automatically monitored and analyzed using the EthoVision tracking system (Noldus Information Technology, Wageningen, Netherlands) (Zhan et al., 2018; Zhang et al., 2018). The open field test was used to test the effect of abdominal surgery on motor activities.

#### TST

Mice were hung individually on the hooks, whose ends were attached to approximately of 2 cm from the tip of the tail for mouse by a small piece of adhesive tape. Immobility time was recorded in 10 min and analyzed by YH-TS behavioral analysis system (Yihong Co., Ltd., Wuhan, China). Mice were considered immobile only when they hung passively or motionless completely. The behavioral performance of mice in TST and FST mimicked hopelessness in trouble of human (Fang et al., 2018; Li et al., 2018).

#### FST

The FST was tested by an automated forced-swim apparatus YH-FST (Yihong Co., Ltd., Wuhan, China). Mice were individually placed in the plastic cylinder (height 31 cm, diameter 23cm), which was filled with water (22~25°C) at a height of 15 cm. Immobility time was recorded in 5 min. Mice were considered immobile if they were floating and no additional movements except for necessary need to keep their heads above the water. The mice were dried gently using a hair dryer and sterilized by iodophor before returning to the cages (Fang et al., 2018; Li et al., 2018).

#### SPT

Mice were exposed to 1% sucrose solution and tap water for 48 h, while exchanging the bottles in the second 24 h to avoid position preference. Followed by 24 h food and water deprivation and an exposure to two identical bottles, one is tap water, and another is 1% sucrose solution. The sucrose and tap water were individually measured by weighing the bottles before and after the test (a 24-h period). The sum of sucrose and tap water were calculated as the total water intake, and the sucrose preference was referred to the percent of sucrose solution intake relative to the total water intake (Fang et al., 2018).

### Western Blot Analysis

Tissues were grated and homogenized with RIPA buffer (Boster, Wuhan, China) at 4°C for 30 min, then centrifuged for 15 min at 4°C. The protein concentrations in supernatants were determined by BCA protein assay kit (Boster, Wuhan, China). The protein samples were separated by 10% sodium dodecyl sulfate–polyacrylamide gel (SDS-PAGE) electrophoresis, and were then transferred to poly vinylidene fluoride (PVDF) membranes(Millipore, Bedford, MA, USA). Bands were blocked with 5% BSA dissolved in TBST (0.1%Tween 20 in Tris-buffered saline) for 1 h at room temperature. Relative primary antibodies were incubated at 4°C overnight: rabbit mBDNF (1:500, DF6387, Affinity, Cincinnati, OH, USA), rabbit TrkB (1:1,000, AF6461, Affinity, Cincinnati, OH, USA), rabbit phosphorylated p-TrkB (1:1,000, AF3461, Affinity, Cincinnati, OH, USA), and rabbit GAPDH (1:2,000, AF7021, Affinity, Cincinnati, OH, USA). After warming and washing by TBST, second antibody was incubated on bands for 2 h at room temperature: goat anti-rabbit IgG horseradish peroxidase (1:5,000, Promotor, Wuhan, China). Finally, these protein bands were visualized by enhanced chemiluminescence substrate solutions (Promotor, Wuhan, China) with the ChemiDoc XRS chemiluminescence imaging system (Bio-Rad, Hercules, CA, USA).

### Statistical Analysis

The data are show as the mean ± standard error of the mean (SEM). Analysis was performed using GraphPad prism software version 7.0. Comparisons among groups were performed using the one-way analysis of variance (ANOVA) or two-way ANOVA, followed by post hoc Tukey test. For different time-points, two-way repeated measures ANOVA were performed to compare the difference among groups. While Fisher's exact tests were used to analyze the proportion of POD susceptible between male and female group or 2 month-old and 16 month-old groups. In hierarchical cluster analysis, the data were firstly standardized by z scores. Then, hierarchical cluster analysis of TST, FST, and SPT was performed using Ward's method and applying squared Euclidean distance as the distance measure, and mice were classified as POD susceptible, resilient, or undetermined clusters. The *P*-values of less than 0.05 were considered statistically significant.

## Results

### Effects of Anesthesia and Surgery on Depression-Like Symptoms in OFT, TST, FST, and SPT Within 1 Month Postoperatively

Within four weeks after anesthesia and surgery, there were no significant differences of total distance traveled in OFT among the groups (**Figure 1B**). TST and FST are classical behavioral observations that detect depressive symptoms and antidepressant effects via evaluation of immobility time in rodents (Yankelevitch-Yahav et al., 2015; Yang et al., 2018). We found TST immobility time was significantly increased on day 1, 15, and 22, but not on day 8, after anesthesia and surgery (**Figure 1C**), while FST immobility time was significantly increased on day 2, 9, 16, and 23 postoperatively (**Figure 1D**). SPT is commonly adopted to assess anhedonia, a core symptom of depression (Yang et al., 2018). In this study, our results demonstrated that SPT scores were significantly decreased on days 6 and 20, but not on 13 and 27, after anesthesia and surgery (**Figure 1E**). In conclusion, the results showed that mice undergone abdominal surgery with isoflurane anesthesia experienced depression-like symptoms in the four weeks following the procedure, with most pronounced effects in the first week.

### Comparisons of Behavioral Results in Control, POD Susceptible, and Resilient Groups Within 1 Week Postoperatively

Mice after anesthesia and surgery were classified into POD susceptible, resilient, and undetermined phenotypes by hierarchical cluster analysis of behavioral results (**Figure 2B**). There were no significant differences in OFT among the groups (**Figure 2C**). Immobility time of TST and FST both showed a significant increase in POD susceptible group as compared with that of control or resilient group (**Figures 2D, E**). Additionally, SPT scores were significantly lower in POD susceptible group than that of control or resilient group (**Figure 2F**).

### BDNF-TrkB Signaling in Brain and Peripheral Tissues in Control, POD Susceptible, and Resilient Group

**Figure 3:**
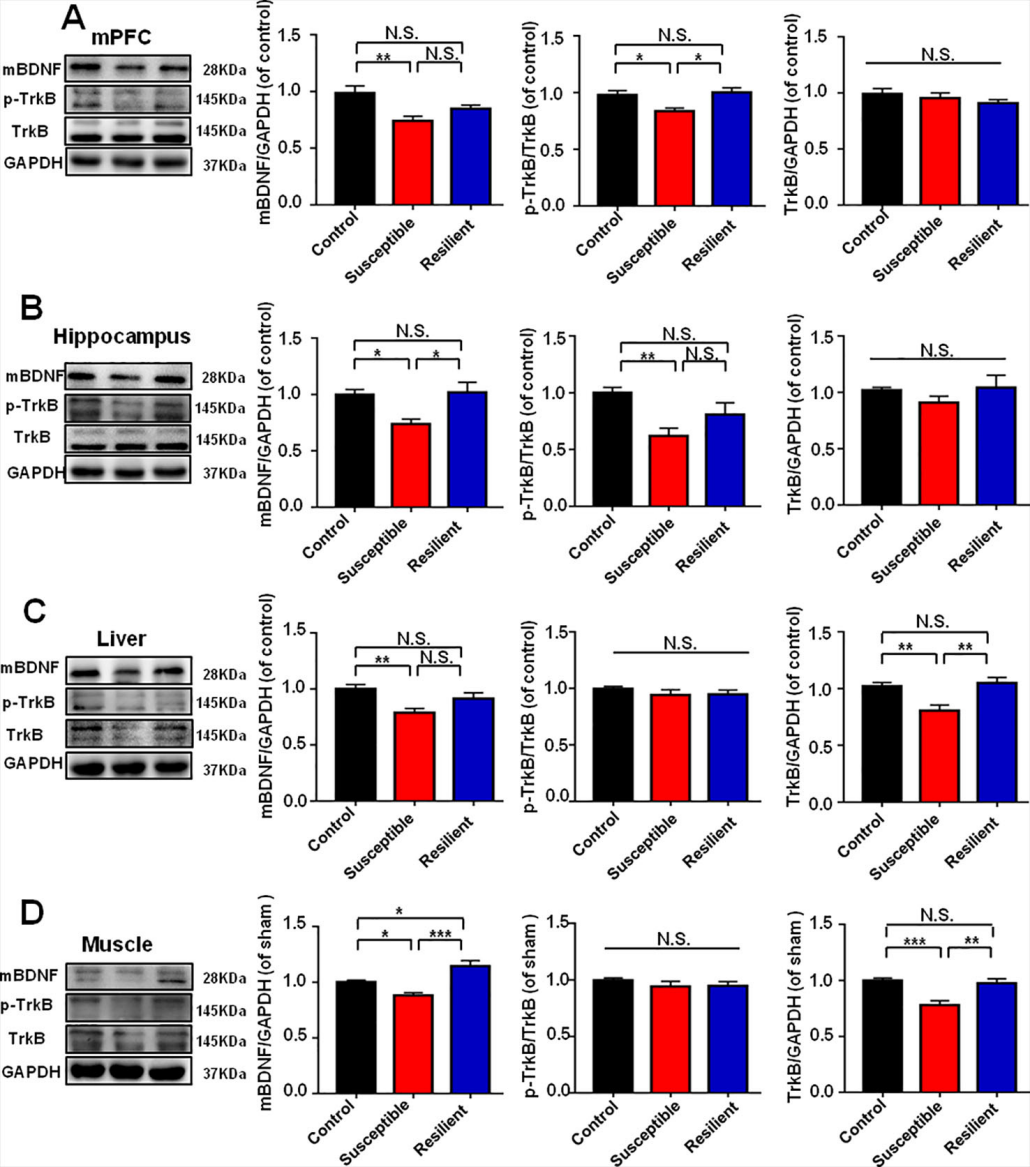
BDNF-TrkB signaling in brain and peripheral tissues in control, POD susceptible, and resilient groups. **(A)** mBDNF [*F*_(2,__17)_ = 6.793, *P* < 0.01], p-TrkB/TrkB [*F*_(2,__17)_ = 6.386, *P* < 0.01] and TrkB [*F*_(2,__17)_ = 0.8234, *P* = 0.4557] levels in the mPFC. **(B)** mBDNF [*F*_(2,__17)_ = 7.019, *P* < 0.01], p-TrkB/TrkB [*F*_(2,__17)_ = 7.011, *P* < 0.001] and TrkB [*F*_(2,__17)_ = 1.063, *P* = 0.3672 levels in the hippocampus. **(C)** mBDNF [*F*_(2,__17)_ = 6.326, *P* = 0.0088], p-TrkB/TrkB [*F*_(2,__17)_ = 0.6828, *P* = 0.5185] and TrkB [*F*_(2,17)_ = 9.607, *P* = 0.0016] levels in the liver. **(D)** mBDNF [*F*_(2,__17)_ = 16.92, *P* < 0.001], p-TrkB/TrkB [*F*_(2,__17)_ = 0.7712, *P* = 0.4780] and TrkB [*F*_(2,__17)_ = 12.65, *P* = 0.0004] levels in the muscle. Data are shown as mean ± SEM (n = 6~7). *P < 0.05, **P < 0.01, or ***P < 0.001. mBDNF, mature brain derived neurotrophic factor; mPFC, medial prefrontal cortex; N.S., not significant; POD, postoperative depression; p-TrkB, phosphorylated tropomyosin-related kinase B; TrkB, tropomyosin-related kinase B.

It has been widely recognized that BDNF-TrkB signaling plays a pivotal role in the pathogenesis and therapeutic mechanisms of depression (Zhang et al., 2014; Shirayama et al., 2015; Yang et al., 2015a; Fang et al., 2018). Mice with POD susceptible and resilient phenotypes both showed a significant decrease in mBDNF levels in mPFC than those in control group (**Figure 3A**). TrkB phosphorylation (p-TrkB)/TrkB ratio was significantly decreased in POD susceptible group as compared with that of control or resilient group (**Figure 3A**). **Figure 3B** depicted that mBDNF level and p-TrkB/TrkB ratio were both decreased in the hippocampus of POD susceptible mice versus control or resilient mice. mBDNF and TrkB levels were measured to be decreased in the liver and muscle of POD susceptible mice than those in control or resilient group. Interestingly, p-TrkB/TrkB ratio failed to show any change among the groups (**Figures 3C, D**).

### Ketamine Treatment Alleviated POD-Like Symptoms and Improved Abnormal Levels of BDNF-TrkB Signaling

**Figure 4:**
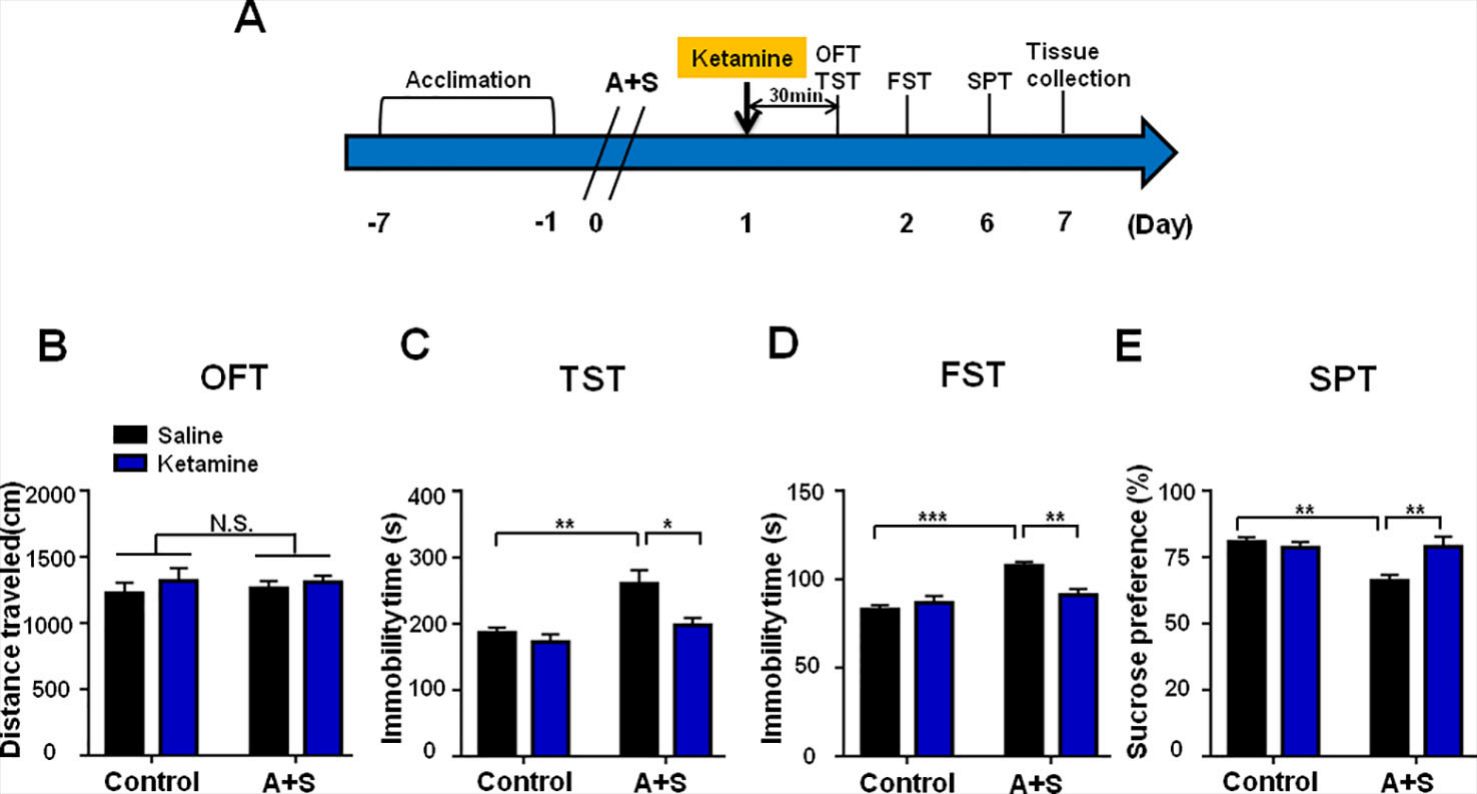
Ketamine pretreatment alleviated POD-like symptoms. **(A)** The schedule of treatment and behavioral tests. Ketamine (10mg/kg, i.p.) was administered on day 1 after A+S, behavioral tests, including OFT, TST, FST, and SPT were performed on day 1, 2, and 5 after A+S, respectively. **(B)** OFT [Group: *F*_(1,__28)_ = 0.032, *P* = 0.8589; Ketamine: *F*_(1,__28)_ = 0.9528, *P* = 0.3374; interaction: *F*_(1,__28)_ = 0.09904, *P* = 0.7553] in control and A+S groups. **(C)** TST [Group: *F*_(1,__28)_ = 13.51, *P* = 0.001; Ketamine: *F*_(1,__28)_ = 7.752, *P* < 0.01; interaction: *F*_(1,__28)_ = 3.116, *P* = 0.0884] was measured in control and A+S groups. **(D)** FST [Group: *F*_(1,__28)_ = 4.124, *P* = 0.0519; Ketamine: *F*_(1,__28)_ = 21.47, *P* < 0.01; interaction: *F*_(1,__28)_ = 10.33, *P* < 0.01] was measured in control and A+S groups. **(E)** SPT [Group: *F*_(1,__28)_ = 4.227, *P* < 0.05; Ketamine: *F*_(1,__28)_ = 7.737, *P* < 0.01; interaction: *F*_(1,__28)_ = 8.566, *P* < 0.01] was measured in control and A+S groups. Data are shown as mean ± SEM (n = 8). *P < 0.05, **P < 0.01, ***P < 0.001. A+S, anesthesia and surgery; FST, forced swimming test; N.S., not significant; OFT, open field test; POD, postoperative depression; SPT, sucrose preference test; TST, tail suspension test.

**Figure 5:**
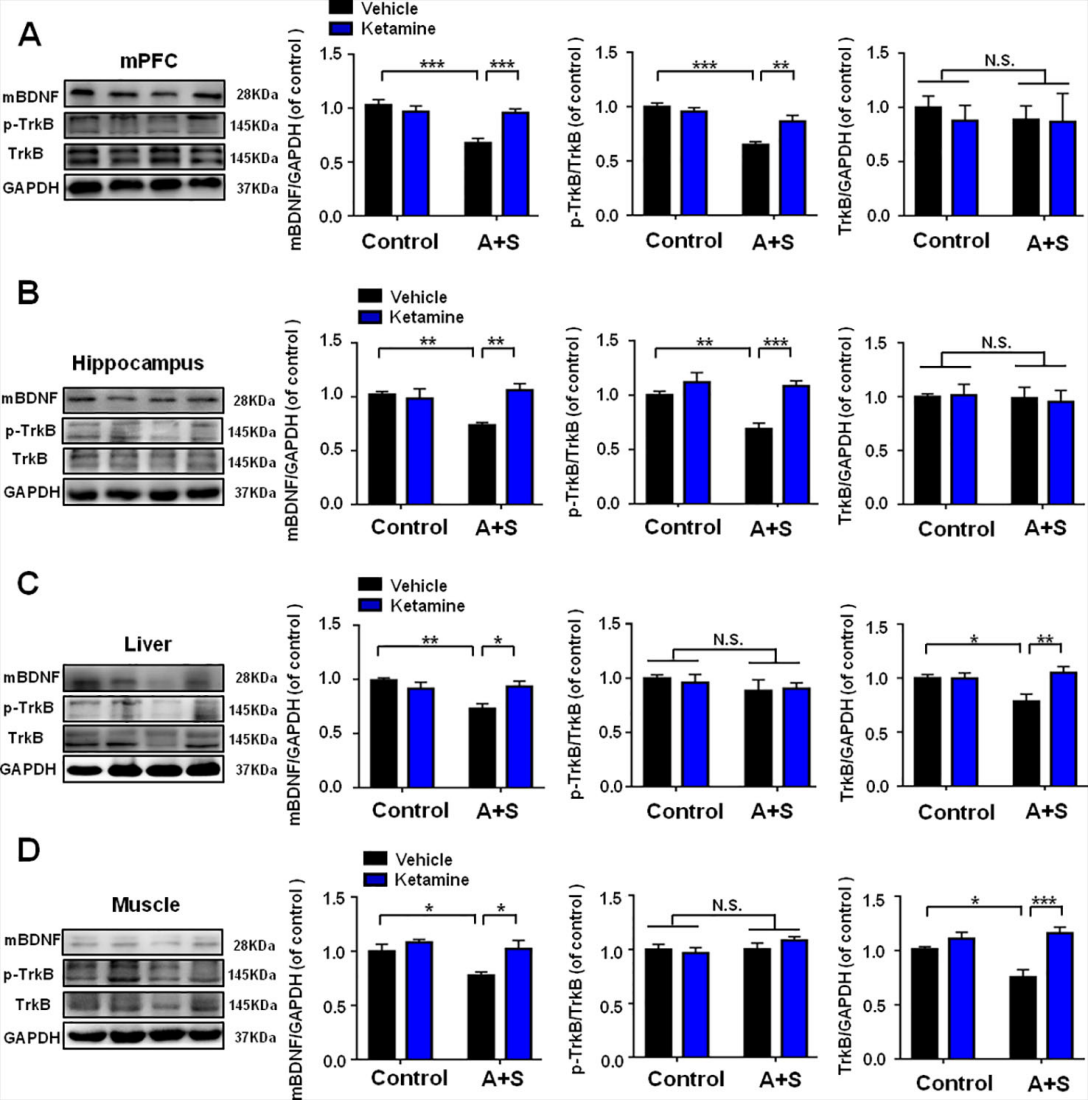
Effects of ketamine pretreatment on BDNF-TrkB signaling in selected tissues. **(A)** mBDNF [Group: *F*_(1,__28)_ = 15.66, *P* < 0.01; Ketamine: *F*_(1,__28)_ = 5.762, *P* < 0.05; interaction: *F*_(1,__28)_ = 14.18, *P* < 0.001], p-TrkB/TrkB [Group: *F*_(1,__28)_ = 29.8, *P* < 0.001; Ketamine: *F*_(1,__28)_ = 4.507, *P* < 0.05; interaction: *F*_(1,__28)_ = 10.55, *P* < 0.01] and TrkB [Group: *F*_(1,__28)_ = 0.1302, *P* = 0.7209; Ketamine: *F*_(1,__28)_ = 0.1722, *P* = 0.6813; interaction: *F*_(1,__28)_ = 0.0913, *P* = 0.7648] levels in mPFC. **(B)** mBDNF [Group: *F*_(1,__28)_ = 3.217, *P* = 0.0837; Ketamine: *F*_(1,__28)_ = 6.182, *P* < 0.05; interaction: *F*_(1,__28)_ = 9.922, *P* < 0.01], p-TrkB/TrkB [Group: *F*_(1,__28)_ = 8.677, *P* < 0.01; Ketamine: *F*_(1,__28)_ = 18.61, *P* < 0.001; interaction: *F*_(1,__28)_ = 5.491, *P* = 0.0265] and TrkB [Group: *F*_(1,__28)_ = 0.1697, *P* = 0.7209; Ketamine: *F*_(1,__28)_ = 0.1697, *P* = 0.6835; interaction: *F*_(1,__28)_ = 0.0787, *P* = 0.7812] levels in the hippocampus. **(C)** mBDNF [Group: *F*_(1,__28)_ = 6.854, *P* < 0.05; Ketamine: *F*_(1,__28)_ = 1.919, *P* = 0.1769; interaction: *F*_(1,__28)_ = 9.254, *P* < 0.01], p-TrkB/TrkB [Group: *F*_(1,__28)_ = 1.571, *P* = 0.2204; Ketamine: *F*_(1,__28)_ = 0.0181, *P* = 0.8938; interaction: *F*_(1,__28)_ = 0.1937, *P* = 0.6632] and TrkB [Group: *F*_(1,__28)_ = 2.286, *P* = 0.1417; Ketamine: *F*_(1,__28)_ = 5.919, *P* < 0.05; interaction: *F*_(1,__28)_ = 6.185, *P* < 0.05] levels in the liver. **(D)** mBDNF [Group: *F*_(1,__28)_ = 8.803, *P* < 0.01; Ketamine: *F*_(1,__28)_ = 8.419, *P* < 0.01; interaction: *F*_(1,__28)_ = 2.964, *P* = 0.0962], p-TrkB/TrkB [Group: *F*_(1,__28)_ = 1.003, *P* = 0.3251; Ketamine: *F*_(1,__28)_ = 1.134, *P* = 0.1961; interaction: *F*_(1,__28)_ = 0.0105, *P* = 0.9192] and TrkB [Group: *F*_(1,__28)_ = 3.494, *P* = 0.0763; Ketamine: *F*_(1,__28)_ = 20.16, *P* = 0.0002; interaction: *F*_(1,__28)_ = 7.58, *P* = 0.0123] levels in the muscle. Data are shown as mean ± SEM (n = 8). *P < 0.05, **P < 0.01, or ***P < 0.001. mBDNF, mature brain derived neurotrophic factor; mPFC, medial prefrontal cortex; N.S., not significant; POD, postoperative depression; p-TrkB, phosphorylated tropomyosin-related kinase B; TrkB, tropomyosin-related kinase B.

Ketamine was administered 30 min before behavioral observations (**Figure 4A**), while failed to show any pharmacological effect on behaviors in OFT (**Figure 4B**). However, two-way repeated measures ANOVA suggested that ketamine could significantly improve abnormal behaviors in TST, FST, and SPT after anesthesia and surgery (**Figures 4C–E**). Importantly, ketamine attenuated decreased mBDNF and p-TrkB/TrkB ratio in mPFC and hippocampus in mice after anesthesia and surgery (**Figures 5A**, **B**). Interestingly, mBDNF and TrkB levels, but not p-TrkB/TrkB ratio, were significantly improved after ketamine administration in mice postoperatively (**Figures 5C, D**).

### 7,8-DHF Treatment Attenuated POD-Like Symptoms

**Figure 6:**
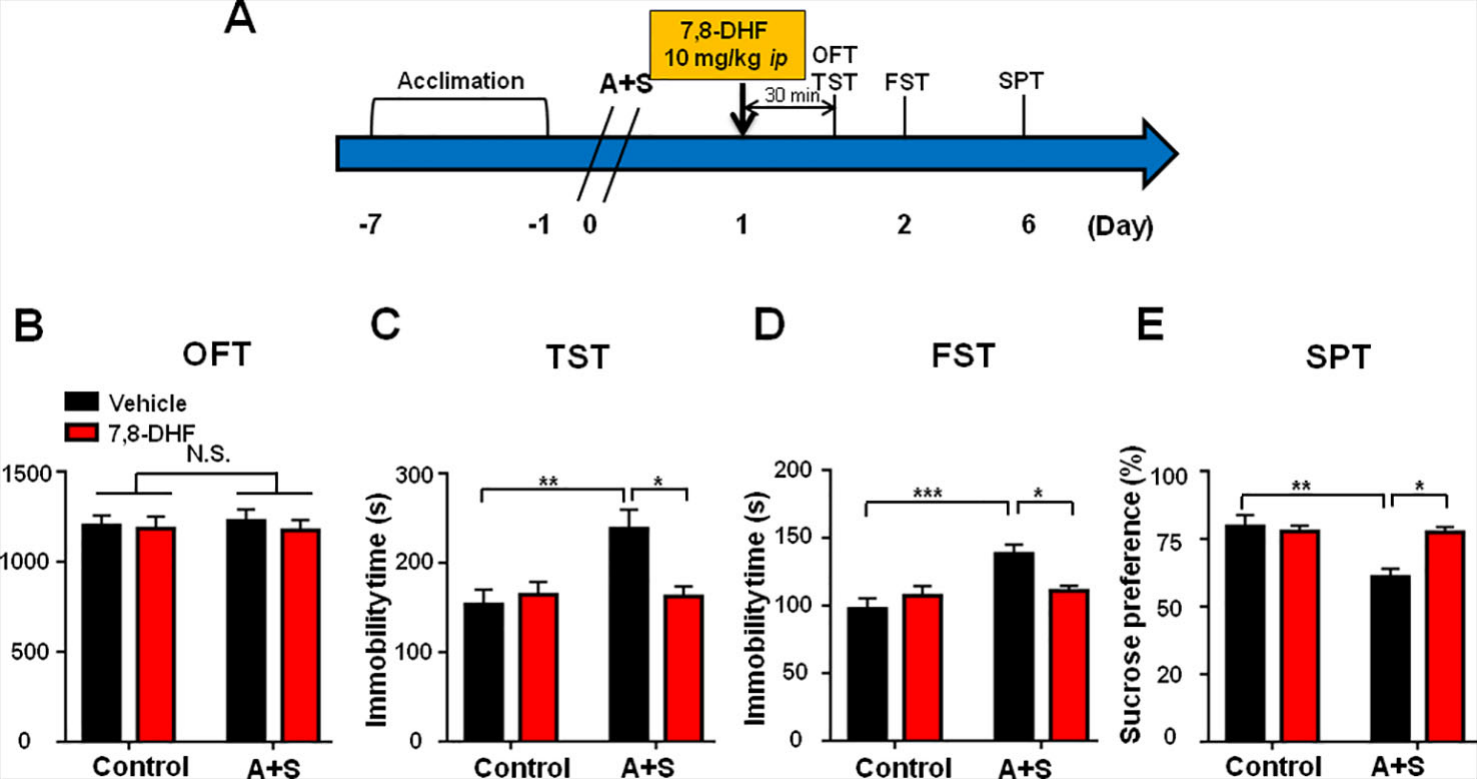
7,8-DHF pretreatment alleviated POD-like symptoms. **(A)** The schedule of treatment and behavioral tests. 7,8-DHF was administered on day 1 after A+S, behavioral tests, including OFT, TST, FST, and SPT were performed on day 1, 2, and 5 after A+S, respectively. **(B)** OFT [Group: *F*_(1,__28)_ = 0.0137, *P* = 0.9075; 7,8-DHF: *F*_(1,__28)_ = 0.3176, *P* = 0.5793; interaction: *F*_(1,__28)_ = 0.0795, *P* = 0.7801]. **(C)** TST [Group: *F*_(1,__28)_ = 6.163, *P* = 0.0196; 7,8-DHF: *F*_(1,__28)_ = 3.817, *P* = 0.0612; interaction: *F*_(1,__28)_ = 6.835, *P* = 0.0144]. **(D)** FST [Group: *F*_(1,__28)_ = 11.07, *P* = 0.0025; 7,8-DHF: *F*_(1,__28)_ = 1.726, *P* = 0.1996; interaction: *F*_(1,__28)_ = 7.659, *P* = 0.0099]. **(E)** SPT [Group: *F*_(1,__28)_ = 9.821, *P* = 0.004; 7,8-DHF: *F*_(1,__28)_ = 5.896, *P* = 0.0218; interaction: *F*_(1,__28)_ = 9.308, *P* = 0.005]. Data are shown as mean ± SEM (n = 8). *P < 0.05, **P < 0.01 or ***P < 0.001. 7,8-DHF: 7,8-dihydroxyflavone; A+S: anesthesia and surgery; FST: forced swimming test; N.S.: not significant; OFT: open field test; POD, postoperative depression; SPT, sucrose preference test; TST, tail suspension test.

7,8-DHF, a potent agonist of BDNF-TrkB signaling, was administered 30 min before behavioral observations (**Figure 5A**). Similarly, it had no effect on distance traveled in OFT (**Figure 5B**). Interestingly, 7,8-DHF significantly attenuated the increased immobility time of TST and FST, and decreased SPT scores in mice (**Figures 6C–E**).

### ANA-12, a BDNF-TrkB Signaling Antagonist, Attenuated Ketamine-Induced Beneficial Effects for POD-Like Symptoms

**Figure 7:**
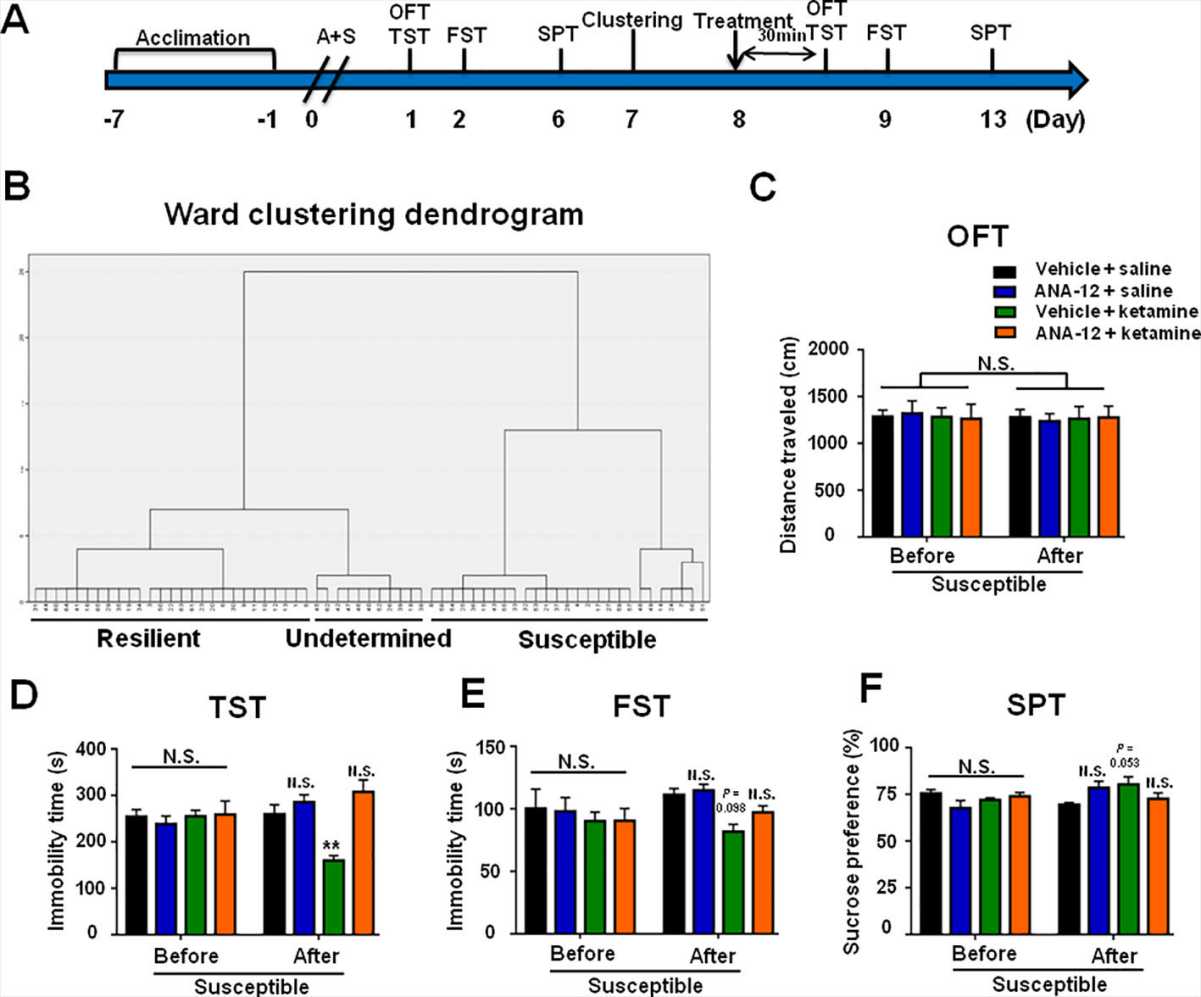
ANA-12 attenuated ketamine-induced beneficial effects on POD-like symptoms in depression susceptible mice. **(A)** The schedule of treatment and behavioral tests. OFT and TST were performed on day 1 and 8 after A+S, respectively; FST was performed on day 2 and 9 after A+S, respectively; SPT was performed on days 6 and 13 after A+S, respectively. Vehicle or saline (10 ml/kg), Ketamine (10 mg/kg), or ANA-12 (0.5 mg/kg) was intraperitoneally injected at a single dose on day 8. **(B)** Dendrogram of hierarchical clustering analysis. A total of 44 mice after A+S were divided into depression susceptible, resilient, and undetermined groups by TST, FST, and SPT results of hierarchical clustering analysis. **(C)** OFT [time: *F*_(1,__20)_ = 0.1232, *P* = 0.7293; group: *F*_(3,__20)_ = 0.0042, *P* = 0.9996; interaction: *F*_(3,__20)_ = 0.09238, *P* = 0.9634]. **(D)** TST [time: *F*_(1,__20)_ = 0.0211, *P* = 0.8859; group: *F*_(3,__20)_ = 3.35, *P* = 0.0396; interaction: *F*_(3,__20)_ = 17.93, *P* < 0.0001]. **(E)** FST [time: *F*_(1,__20)_ = 1.67, *P* = 0.211; group: *F*_(3,__20)_ = 1.878, *P* = 0.1659; interaction: *F*_(3,__20)_ = 1.16, *P* = 0.3497]. **(F)** SPT [time: *F*_(1,__20)_ = 1.856, *P* = 0.1882; group: *F*_(3,__20)_ = 0.7368, *P* = 0.5424; interaction: *F*_(3,__20)_ = 3.35, *P* = 0.0396]. Data are shown as mean ± S.E.M. (n = 6 per group). **P < 0.01 A+S: anesthesia and surgery; ANA-12: N-[2-[[(Hexahydro-2-oxo-1H-azepin-3-yl)amino]carbonyl] phenyl]-benzo[b] thiophene-2-carboxamide; FST, forced swimming test; N.S., not significant; OFT, open field test; POD, postoperative depression; SPT, sucrose preference test; TST, tail suspension test.

Mice with POD susceptible phenotype were selected by hierarchical cluster analysis of behaviors on day 7 postoperatively (**Figures 7A, B**). OFT showed no statistical differences in the distance traveled of mice before and after drugs treatment. However, ANA-12 significantly attenuated the facilitating effects of ketamine on POD-like symptoms in susceptible mice (**Figures 7D–F**).

### Roles of Sex and Age Differences in POD-Like Symptoms

**Figure 8:**
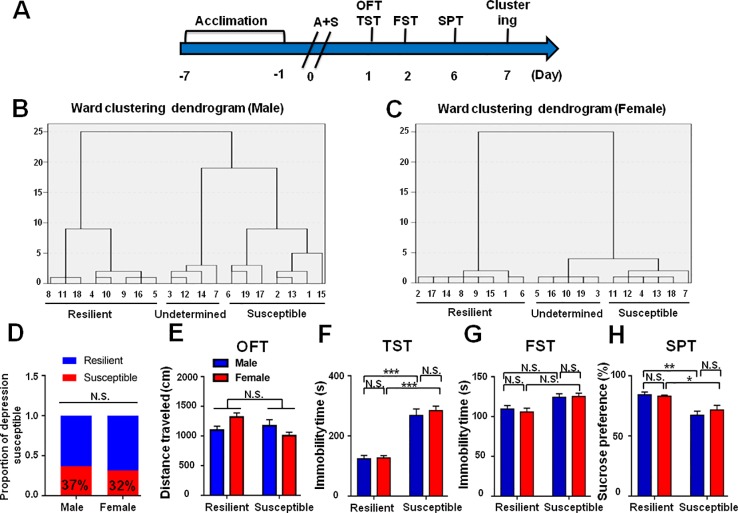
Role of sex deference in POD-like symptoms. **(A)** The schedule of this study. A+S was performed on day 0 after acclimation. Behavioral tests, including OFT, TST, FST, and SPT were performed on days 1, 2, and 5 after A+S, respectively. **(B)** Dendrogram of hierarchical clustering analysis. A total of 19 mice were divided into POD susceptible, resilient, and undetermined phenotypes by TST, FST, and SPT results. Seven of 19 mice in male group were POD susceptible phenotype. **(C)** Dendrogram of hierarchical clustering analysis. A total of 19 mice were divided into POD susceptible, resilient, and undetermined phenotypes by TST, FST, and SPT results. Six of 19 mice were POD susceptible phenotype in female group. **(D)** The ratios of POD susceptible mice in male and female groups were 37 and 32% (Fisher's exact tests, *P* > 0.9999), respectively. **(E)** OFT [Phenotype: *F*_(1,__23)_ = 1.193, *P* = 0.1779; Sex: *F*_(1,__23)_ = 0.2481, *P* = 0.6231; interaction: *F*_(1,__23)_ = 5.514, *P* < 0.05]. **(F)** TST [Phenotype: *F*_(1,__23)_ = 98.32, *P* < 0.001; Sex: *F*_(1,__23)_ = 0.5517, *P* = 0.4651; interaction: *F*_(1,23)_ = 0.321, *P* = 0.5765]. **(G)** FST [Phenotype: *F*_(1,__23)_ = 12.4, *P* < 0.05; Sex: *F*_(1,__23)_ = 0.5007, *P* = 0.4863; interaction: *F*_(1,__23)_ = 0.0209, *P* = 0.8861]. **(H)** SPT [Phenotype: *F*_(1,__23)_ = 26.42, *P* < 0.001; Sex: *F*_(1,__23)_ = 0.0796, *P* = 0.7804; interaction: *F*_(1,__23)_ = 0.4816, *P* = 0.4946]. Data are shown as mean ± S.E.M. (n = 6~7). *P < 0.05, **P < 0.01, or ***P < 0.001. A+S, anesthesia and surgery; FST, forced swimming test; N.S., not significant; OFT, open field test; POD, postoperative depression; SPT, sucrose preference test; TST, tail suspension test.

**Figure 9:**
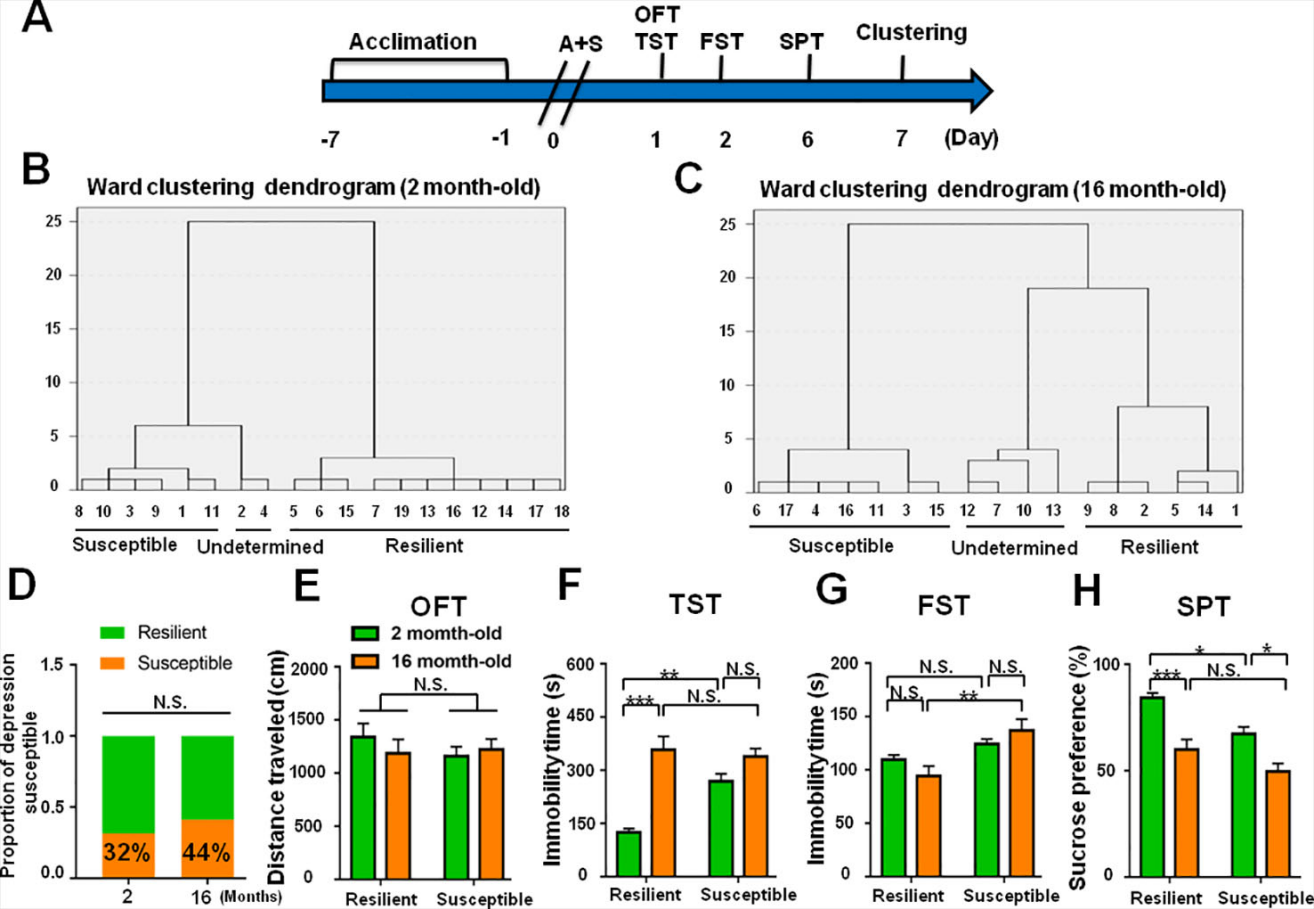
Role of age deference in POD-like symptoms. **(A)** The schedule of this study. A+S was performed on day 0 after acclimation. Behavioral tests, including OFT, TST, FST, and SPT were performed on day 1, 2, and 5 after A+S, respectively. **(B)** Dendrogram of hierarchical clustering analysis. A total of 19 mice were divided into POD susceptible, resilient, and undetermined phenotypes by TST, FST, and SPT results. Six of 19 mice in 2 month-old group were POD susceptible phenotype. **(C)** Dendrogram of hierarchical clustering analysis. A total of 16 mice were divided into POD susceptible, resilient, and undetermined phenotypes by TST, FST, and SPT results. Seven of 16 mice were POD susceptible phenotype in 16 month-old group. **(D)** The ratios of depression-susceptible mice in 2 month-old and 16 month-old groups were 32% and 44% (Fisher's exact tests, *P* = 0.5031), respectively. **(E)** OFT [Phenotype: *F*_(1,__22)__=_ 0.3991, *P* = 0.5341; Age: *F*_(1,__22)_ = 0.1418, *P* = 0.7101; interaction: *F*_(1,__22)_ = 0.2509, *P* = 0.6214]. **(F)** TST [Phenotype: *F*_(1,__22)_ = 7.521, *P* = 0.0119; Age: *F*_(1,__22)_ = 37.1, *P* < 0.0001; interaction: *F*_(1,22)_ = 12.37, *P* < 0.05]. **(G)** FST [Phenotype: *F*_(1,__22)_ = 14.46, *P* = 0.001; Age: *F*_(1,__22)_ = 0.1098, *P* = 0.7435; interaction: *F*_(1,__22)_ = 2.687, *P* = 0.1154]. **(H)** SPT [Phenotype: *F*_(1,__22)_ = 12.78, *P* < 0.01; Age: *F*_(1,__22)_ = 32.66, *P* < 0.0001; interaction: *F*_(1,__22)_ = 0.6142, *P* = 0.4416]. Data are shown as mean ± S.E.M. (n = 6~7). *P < 0.05, **P < 0.01, or ***P < 0.001. A+S, anesthesia and surgery; FST, forced swimming test; N.S., not significant; OFT, open field test; POD, postoperative depression; SPT, sucrose preference test; TST, tail suspension test.

The incidence of POD in female mice was slightly lower than that of male mice, although there was no statistically significant difference (**Figures 8A–H**). In addition, it has been reported that depression has a higher incidence in the elderly individuals. We demonstrated that the incidence of POD in aged mice is 44%, which is higher than young subjects (**Figures 9A–H**).

## Discussion

A postoperative depression model was utilized to reveal that anesthesia and surgery induce depression-like phenotypes in the four weeks following the procedure, with most pronounced effects in the first week. After anesthesia and surgery, approximately 30% of mice showed POD-like symptoms via hierarchical cluster analysis of behavioral results. Levels of BDNF-TrkB signaling were significantly lower in brain and peripheral tissues in POD susceptible mice than those in control or resilient group. Ketamine administered 30 min before behavioral tests restored abnormalities in behavioral performance in TST, FST, and SPT, and associated with improvements in levels of BDNF-TrkB signaling. Furthermore, the beneficial effects of ketamine on POD are pharmacologically blocked by TrkB antagonist ANA-12. To the best of our knowledge, this is the first study demonstrating abnormal levels of BDNF-TrkB were implicated in POD. This is also the first study reporting role of BDNF-TrkB signaling in the beneficial effects of ketamine on POD.

Given the possibility that the surgery-related limitations on physical abilities could influence the results of TST and FST at early time-points. We have noticed the influence of surgery-related discomfort on the results of TST and FST, a tiny incision followed by the three-day postoperative analgesia was applied to alleviate the negative effects on behavioral tests. The mechanical threshold withdraw test for three days postoperatively shown that there was no significant difference between the control and A+S groups (Not shown in this study). Furthermore, hierarchical cluster analysis of TST, FST, and SPT results was performed to classify the A+S mice into depression susceptible and resilient groups, but we found an interesting phenomenon that the clustering result of the three tests was similar to that of SPT solely. After checking the original data, we concluded that SPT results were more constant than the others, suggesting SPT may be a major factor of the clustering results. Next, our previous study (Zhang et al., 2019) indicated that postoperative delirium was at high risk during early-stage after surgery and anesthesia, in which emotional disorder, such as depression, occurred frequently in clinics (Samuelsson et al., 2007; Costigan et al., 2009; Patel and Sun, 2009). Hence, behavioral tests at early time-points were suitable to be employed to observe the depression-like symptoms in mice.

It has been commonly known that the role of BDNF-TrkB signaling in the pathogenesis and treatment mechanisms of depression (Zhang et al., 2014; Shirayama et al., 2015; Yang et al., 2015a; Fang et al., 2018). We previously reported that lower levels of BDNF-TrkB signaling in mPFC and hippocampus might contribute to the onset of stress susceptible in LH and CSDS models (Yang et al., 2015a; Yang et al., 2015b; Yang et al., 2016). In this study, the results demonstrated that BDNF-TrkB signaling in mPFC and hippocampus was significantly lower in POD susceptible mice than those in control or POD resilient group, consistent with our previous results. Intriguingly, accumulating evidence is concerned with the role of peripheral tissues, particularly liver and muscle, in the pathogenesis of depression (Yang et al., 2016). We found in this study that BDNF and TrkB levels were both significantly decreased in liver and muscle of susceptible subjects in the POD model. It is currently believed that physical exercise improves depression symptoms may be related to the upregulation of BDNF in brain via exercise-stimulated BDNF release in muscle (Heyman et al., 2012; Yang and Luo, 2017). Does this mean that BDNF in muscle improved by exercise could be helpful to prevent and treat POD? Further large-scale clinical trials are required to validate this finding.

As mentioned previously, ketamine has a rapid and long-lasting antidepressant effect in both MDD and BD (Grunebaum et al., 2017; Vande et al., 2017; Kadriu et al., 2018). Our results in this study suggest that ketamine also has a significant improvement in POD, and this beneficial effect could be totally attenuated by TrkB antagonist ANA-12, suggesting that BDNF-TrkB signaling plays an important role in ketamine exerting antidepressant effects in POD. Not only are the antidepressant effects of ketamine associated with activation of the BDNF-TrkB signaling pathway, but also the antidepressant effects of R-, S-ketamine, and S-norketamine are both related to the up-regulation of BDNF-TrkB signaling (Yang et al., 2015a; Yang et al., 2018). As previously reported, a dose of ketamine at 10mg/kg can result in acute (1 h) and long-lasting (24 h) dose-dependent antidepressant effects in mice (Zanos et al., 2016). The acute antidepressant effects of ketamine are due to inhibition of the N-methyl-D-aspartic acid receptor (NMDAR) by ketamine and its N-demethylated metabolite (R,S)-norketamine. Nevertheless, the sustained (lasting for at least 3 days) antidepressant effects in the FST are associated with ketamine metabolite, (2R,6R)-hydroxynorketamine, via the upregulation of α-amino-3-hydroxy-5-methyl-4-isoxa-zolep-propionate receptors (AMPARs) in hippocampus. In this regard, we inferred that ketamine isomers and metabolites may have therapeutic effects on POD. It is therefore likely that the use of small doses of ketamine during the surgery could not only enhance the pharmacological efficacy of anesthetics, but also effectively prevent and treat POD.

It has been recognized that age and sex differences are the triggering factors in the incidence of depression and postoperative complications (Luppa et al., 2012). In this study, sex and age differences failed to show statistical changes impacting the POD incidence although there was a slight difference between the groups. Further studies on the roles of age and sex differences in the onset and pathogenesis of POD are exceedingly required.

In conclusion, abnormality in BDNF-TrkB signaling in brain and peripheral tissues might contribute to the onset of POD, and that ketamine-elicited improving effects on POD are likely related to the up-regulation of BDNF-TrkB signaling. Further large-scale clinical studies on the beneficial effects of ketamine on POD are exceedingly needed.

## Data Availability Statement

The raw data supporting the conclusions of this manuscript will be made available by the authors, without undue reservation, to any qualified researcher.

## Ethics Statement

The animal study was reviewed and approved by the Experimental Animal Committee of Tongji Hospital, Tongji Medical College, Huazhong University of Science and Technology.

## Author Contributions

SL, CY, and AL designed this study and wrote the protocol and the manuscript. SL, XL, DY, YW, and GZ performed the experiments. SL and CY conducted the statistical analysis. XL revised the manuscript. All the authors contributed to this study and approved the final manuscript and this submission.

## Funding

This study was supported by grants from the National Natural Science Foundation of China (No.: 81771159, 81571047, 81703482, 81974160, and 81974171), “333 Project” of Jiangsu Province (No.: BRA2016122), Program of Bureau of Science and Technology Foundation of Changzhou (No: CJ20159022, CJ20160030 and CJ20179028), and Major Science and Technology Projects of Changzhou Municipal Committee of Health and Family Planning (No: ZD201505 and ZD201407).

## Conflict of Interest

CY received the research support from B. Braun Medical Inc.

The remaining authors declare that the research was conducted in the absence of any commercial or financial relationships that could be construed as a potential conflict of interest.
